# Establishing a health demographic surveillance site in Bhaktapur district, Nepal: initial experiences and findings

**DOI:** 10.1186/1756-0500-5-489

**Published:** 2012-09-05

**Authors:** Umesh Raj Aryal, Abhinav Vaidya, Suraj Shakya-Vaidya, Max Petzold, Alexandra Krettek

**Affiliations:** 1 Dept of Community Medicine, Kathmandu Medical College, Kathmandu, Sinamangal, Nepal; 2 Nordic School of Public Health NHV, Box 12133, 402 42, Gothenburg, Sweden; 3 Department of Ophthalmology, Nepal Medical College, Kathmandu, Jorpati, Nepal; 4 Center for Applied Biostatistics, Sahlgrenska Academy at University of Gothenburg, Gothenburg, Sweden; 5 Department of Internal Medicine and Clinical Nutrition, Institute of Medicine, Sahlgrenska Academy at University of Gothenburg, Gothenburg, Sweden

## Abstract

**Background:**

A health demographic surveillance system (HDSS) provides longitudinal data regarding health and demography in countries with coverage error and poor quality data on vital registration systems due to lack of public awareness, inadequate legal basis and limited use of data in health planning. The health system in Nepal, a low-income country, does not focus primarily on health registration, and does not conduct regular health data collection. This study aimed to initiate and establish the first HDSS in Nepal.

**Results:**

We conducted a baseline survey in Jhaukhel and Duwakot, two villages in Bhaktapur district. The study surveyed 2,712 households comprising a total population of 13,669. The sex ratio in the study area was 101 males per 100 females and the average household size was 5. The crude birth and death rates were 9.7 and 3.9/1,000 population/year, respectively. About 11% of births occurred at home, and we found no mortality in infants and children less than 5 years of age. Various health problems were found commonly and some of them include respiratory problems (41.9%); headache, vertigo and dizziness (16.7%); bone and joint pain (14.4%); gastrointestinal problems (13.9%); heart disease, including hypertension (8.8%); accidents and injuries (2.9%); and diabetes mellitus (2.6%). The prevalence of non-communicable disease (NCD) was 4.3% (95% CI: 3.83; 4.86) among individuals older than 30 years. Age-adjusted odds ratios showed that risk factors, such as sex, ethnic group, occupation and education, associated with NCD.

**Conclusion:**

Our baseline survey demonstrated that it is possible to collect accurate and reliable data in a village setting in Nepal, and this study successfully established an HDSS site. We determined that both maternal and child health are better in the surveillance site compared to the entire country. Risk factors associated with NCDs dominated morbidity and mortality patterns.

## Background

The current estimation of disease burden in Nepal is based on cross-sectional studies, including the National Census and studies conducted by the Ministry of Health and other agencies. These health data lack complete epidemiological information to support critical decisions by health planners, policymakers and health managers. Additionally, Nepal’s registration systems for vital statistics include coverage errors (e.g., only 35% of live births are registered and no causes of deaths were available) due to lack of public awareness, inadequate legal basis and limited use in health planning and policy [1,2]. Proper planning and budget allocations for appropriate preventive measures require reliable, accurate and continuous information about community-level health issues [3-5].

A Health Demographic Surveillance System (HDSS) provides a method for collecting data, based on longitudinal measurement of population dynamics, health and social changes, to inform and advocate public health policymakers and practitioners [6]. Such surveillance sites are restricted to a specified geographic area, and data are collected at regular intervals. Many low- and middle-income countries in Asia and Africa have already established HDSS under INDEPTH, an international network of field sites, thus providing continuous demographic evaluation of population and health in developing countries [7]. By undertaking population-level research, HDSS also engender a thorough understanding of disease burden. Using interventional studies, HDSS can also be useful in studying specific public health strategies [8,9].

The overall aim of an HDSS involves developing an epidemiological surveillance site to produce basic population-based health data; serving as a background and sampling frame for specific studies, especially longitudinal studies; creating formal training capabilities, particularly for epidemiological training of research students; and providing evidence to policy-makers to support better policies and healthcare interventions [9,10].

Nepal currently lacks the capacity to provide reliable and accurate data collection on a longitudinal basis. Therefore, this study aimed to establish an HDSS in Bhaktapur district as a collaborative effort between the Nordic School of Public Health NHV, Sweden; Kathmandu Medical College (KMC) and Nepal Medical College (NMC), Nepal. The HDSS concept in Nepal is still in the initial stage of development therefore we encountered many challenges related to practical issues around supplying imperative information for health planning and interventions. Here, we describe the preliminary experiences and findings of the newly established HDSS in Jhaukhel-Duwakot, a village area of Nepal.

## Methods

### Study site and population

Duwakot and Jhaukhel villages lie in the mid-hills of Bhaktapur district, 13 kilometers outside the capital city, Kathmandu. These villages were chosen for HDSS primarily due to their proximity to the community hospitals run by KMC and NMC and secondarily due to them being prototypical urbanizing villages by the larger towns in Nepal.

**Figure 1:**
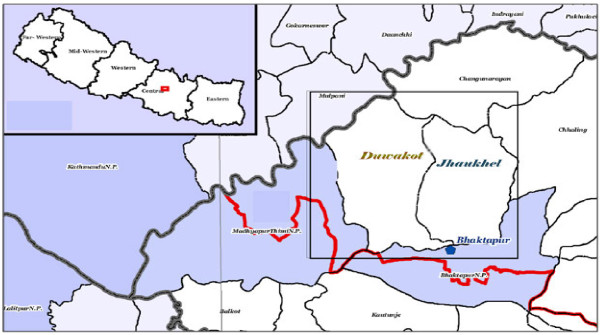
**Map of Nepal showing Bhaktapur district (insert) and the location of the Health Demographic Surveillance Site (HDSS) in Duwakot and Jhaukhel villages in Bhaktapur district (right) **[11].

Duwakot is located 1,367 meters above sea level and has an area of 6.42 square kilometers (Figure 1) [11]. KMC operates a 70-bed community hospital in this village. Adjacent to the east of Duwakot, Jhaukhel is 1,401 meters above sea level and covers an area of 5.41 square kilometers. NMC operates a 25-bed community hospital in this village. The national government maintains a health post in each of the two villages.

Duwakot and Jhaukhel share a common geography, ethnicity and culture, and concrete roads provide both villages with accessibility to the Bhaktapur-Kathmandu Highway. Most households in Duwakot and Jhaukhel have piped water, electricity and modern communication facilities. Although both villages maintain primary and secondary schools, they lack high schools or colleges other than the medical colleges.

### Manpower recruitment

A core local management committee, consisting of an HDSS coordinator and PhD students, was formed to oversee HDSS activities. Every village in Nepal is divided administratively into nine wards. We recruited 18 female enumerators (1 per ward) for data collection all enumerators lived in the same ward where they would collect data. We recruited female enumerators mainly for better compliance and cooperation as the respondents are culturally more receptive to them. The minimum qualification required to be enumerator was completion of grade 10. We also hired two male field supervisors, one for each village.

### Household listing and social mapping

In August 2010, the surveyors were trained to (i) properly list and enumerate the households in each ward and (ii) prepare a manually drawn social map for each village. The surveyors then pretested the questionnaire in the nearby houses, emphasizing informed consent; each respondent was free to participate or end the interview at any time. Finally, trained data entry operators created a database of the list, using Epidata 3.1 software.

### Tools

The baseline census questionnaire was developed using the FilaBavi and DodaLab HDSS model that was developed in Viet Nam [12]. The experience of management committee members in the Community Diagnosis (CD) Programmes of the medical colleges helped tailor the questionnaire to the local Nepali context. The questionnaire contained semi-structured questions on demographic parameters, such as birth, death, marriage and migration; health and health-seeking behaviours; and social, environmental and economic factors. Each question was coded for data entry. After completing the household listing, the enumerators were trained in baseline data collection, which was conducted between October and December 2010.

The socioeconomic class was defined by Kuppuswamy’s socioeconomic status scale modified to the Nepalese context [13]. The scale takes into account the education status, occupation and family income per month to categorize the family into high, middle and low socio economic status. The income classification of the original scale in Indian currency was here converted into Nepalese Rupees by multiplying with 1.6 (1 Rs Indian = 1.60 Rs. Nepali) and also using the 2010 consumer price index of Nepal. The Reliability Coefficient (α) was 0.503 in the current study.

Illness was recorded for 4 weeks immediately preceeding the survey. Attrition of death was done on the basis of the response provided by the respondent. Multiple responses were recorded if more than one cause was provided for both morbidity and mortality.

### Monitoring, supervision, and quality control

Field supervisors, the HDSS coordinator and the PhD students supervised the collection process on a regular basis, and supervisors oversaw the interview process. To double-check the quality of data collection, the supervisors also conducted random, repeat interviews in 5% of the households. To rectify problems encountered in the field during data collection and to obtain maximum response and reliable data from the community, enumerators, supervisors and PhD students held regular discussions.

### Data management and analysis

We outsourced both data entry of the household listings and baseline data to a team of public health graduates, who entered it into Epidata software, version 3.1. We checked the data entry process regularly, seeking feedback and discussion about the many unforeseen problems as they arose (see below). Data analysis was conducted using Microsoft Excel 2007; SPSS Statistics 17.0; and STATA 10. We used both descriptive (percentage, mean, standard deviation) and inferential statistics (logistic regression) for data analysis. Besides these, we also computed different types of crude and specific rates and ratios.

### Ethical issues

We obtained informed verbal consent from all respondents. Ethical approval was obtained from Nepal Health Research Council (NHRC). We also briefed local administrative authorities, health personnel and political leaders about the study’s objectives, and we obtained their verbal permission to conduct the survey. To ensure confidentiality, all data were secured in the HDSS office. The participating Nepalese medical institutes provided subsidized medical services to respondents who required care during the survey.

## Results

**Figure 2:**
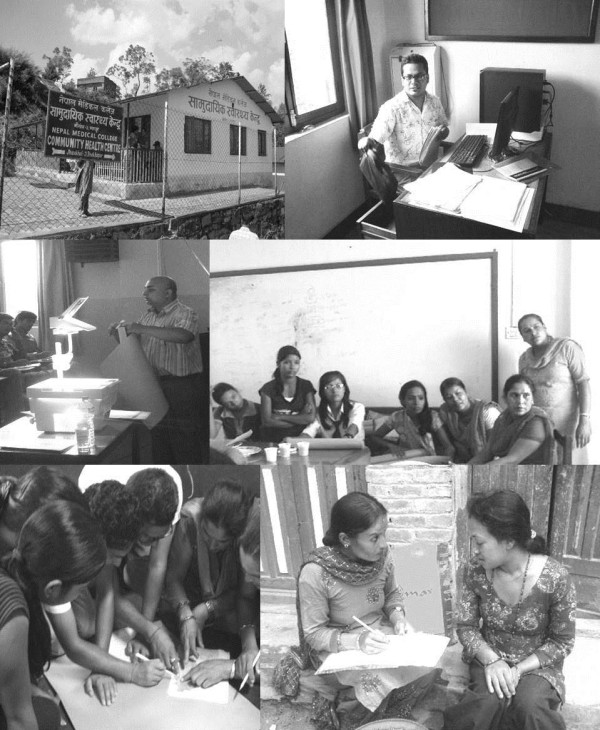
Photos (from top left to lower right): HDSS office (Nepal Medical College, Jhaukhel) and the office room; training session; enumerators during training; the making of social maps; enumerator during an interview (baseline survey training and action).

In 2010, the Jhaukhel-Duwakot Health Demographic Surveillance Site (JD-HDSS) was established in Nepal with its main office in the NMC community hospital in Jhaukhel (Figure 2).

### Findings of the baseline census 2010

**Figure 3:**
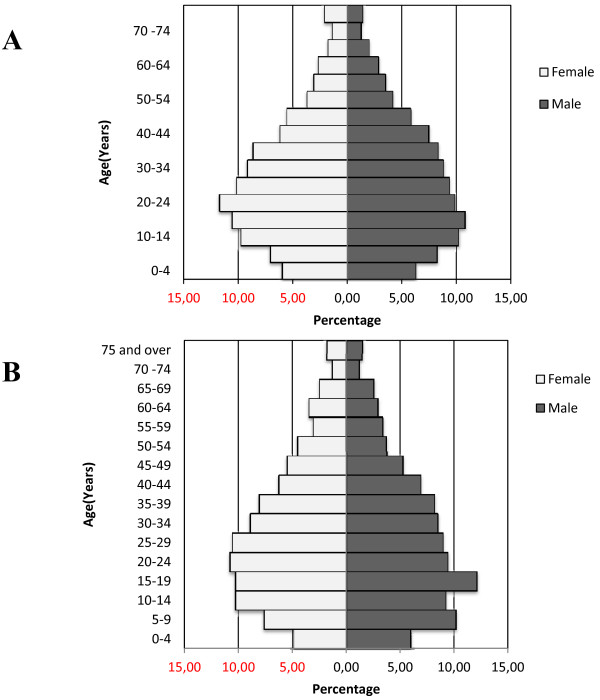
Population pyramid of the Duwakot (A) and Jhaukhel (B) development committees, Baseline Survey 2010.

Figure 3 and Tables 1, 2, 3 present the findings, separately and combined, of the 2010 baseline census. JD-HDSS includes 2,712 households containing a total of 13,669 individuals (Table 1). The study population covers nearly 5% of the total population of Bhaktapur district [14]. The total population of the study area has increased nearly by 3% (annual growth rate = 0.26%) and total household number has increased by 15% after the national census 2001 [15]. The average household size of JD-HDSS is similar to the data reported by the Nepal Health Demographic Survey 2006 [16]. Households in both villages showed a 96% response rate during data collection. With few exceptions, all forms were filled in completely.

**Table 1:** Household size, population size and sex ratio of JD-HDSS, Bhaktapur, 2010

**Variable**	**Duwakot**	**Jhaukhel**	**Total**
Total households	1,557	1,155	2,712
Total population	7,612	6,057	13,669
Males	3,819	3,049	6,868
Females	3,793	3,008	6,801
Sex ratio (male per female)	1.001	1.010	1.010
Median household size (range)	5.0 (1–16)	5.0 (1–21)	5.0 (1–21)

**Table 2:** Vital statistics, JD-HDSS, Bhaktapur, 2010

**Variable**	**Duwakot**	**Jhaukhel**	**Total**
**Fertility**			
Crude birth rate (per 1,000 population)	12.2	6.6	9.7
General fertility rate (per 1000 female population, 15–49 yrs)	39.4	22.3	32
Sex ratio at birth (male per female)	1.16	1.11	1.15
Child: Women ratio (per 1,000 female population, 15–49 yrs)	194.1	177.9	187.2
Home delivery (%)	12.9	7.7	11.3
**Mortality**			
Crude death rate (per 1,000 population)	4.3	3.5	3.9
Infant mortality rate (per 1,000 live births)			
Maternal mortality ratio (per 1,000,000 live births)			
Premature death (under 65 years) (%)	24.2	42.8	31.5
Death registration (%)	75.8	52.4	66.7
**Morbidity**			
Illness (%)	7.9	13.2	11.1
**Migration**			
In-migration (%)	3.42	0.79	2.25
Out-migration (%)	1.16	1.61	1.36

Infant mortality rate and maternal mortality ratio are not shown as there were no deaths reported. Illness was recorded for 4 weeks immediately preceeding the survey. Out-migration did not cover the families in which all the family members of the household had migrated.

**Table 3:** Common causes of mortality, JD-HDSS, Bhaktapur, 2010

**Causes of deaths**	**Duwakot**	**Jhaukhel**	**Total**
	**Number**	**Number**	**Number**
	**(N = 33)**	**(N = 21)**	**(N = 54)**
Old age	9	8	17
Respiratory diseases	7	2	9
Stroke	6	1	7
Diabetes mellitus	4	2	6
Malignant neoplasm (cancer)	0	5	5
Gastrointestinal diseases	0	2	2
Genito-urinary diseases	1	1	2
Suicide	2	0	2
Hypertension	1	1	2
Inconclusive/not sure	2	1	3

Data is for deaths that had occurred in the preceeding year before the survey. Attrition of death was done on the basis of the response provided by the respondent. Multiple responses were recorded if more than one cause was provided. Respiratory diseases included both chronic obstructive lung diseases and pulmonary infections.

### Socio-demographic findings

According to the age-sex pyramids of the two villages, a majority of the population was between 10 and 30 years of age, and the birth rate was low (Figure 3A and 3B). The proportion of population is decreasing with age group after 20–24 years. It indicates that the proportion of deaths may rise in the future as a consequence of low birth rate. The median age of the population is 27 years for both males and females.

**Figure 4:**
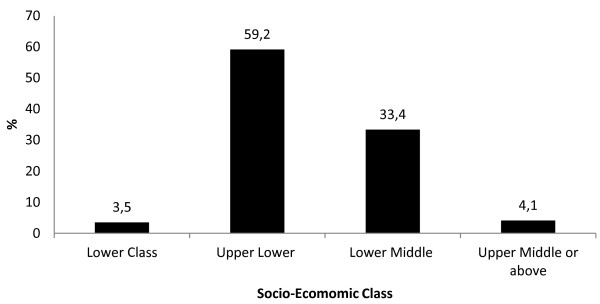
**Socio-economic classification of the households in the Health Demographic Surveillance site at Jhaukhel and Duwakot (JD-HDSS).** The socioeconomic class was defined by Kuppuswamy’s socioeconomic status scale modified to the Nepalese context [13]. The scale takes into account the education status, occupation and family income per month to categorize the family into high, middle and low socio economic status. The income classification of the original scale in Indian currency was here converted into Nepalese Rupees by multiplying with 1.6 (1 Rs Indian = 1.60 Rs. Nepali) and also using the 2010 consumer price index of Nepal. The Reliability Coefficient (α) was 0.503 in the current study.

The predominant castes were Newars (36.5%), Chhetris (30.4%) and Brahmins (23.4%); and nearly 97% of the population was Hindu. Among employable individuals, 25% were students aged between 10 and 30 years, fewer than 11% worked in agriculture, nearly 20% were service holders, and 2% were unemployed. Almost one fifth (18.2%) of the individuals ≥ 6 years of age were illiterate. More than two thirds of the population was economically active and based on education, occupation and income levels attained by heads of the households, about 60% belonged to the upper-lower class (Figure 4); 93% owned their own home. Around 50% of households used piped drinking water, almost all (99.5%) had electricity, and 3% lacked indoor toilet facilities.

### Fertility-related findings

Fertility–related indicators for JD-HDSS offered a good picture of overall fertility rate, with a crude birth rate of 9.7/1,000 total population and a home delivery rate of 11% (Table 2). Mean age at marriage (± standard deviation) was 22.2 (±4.6) and 18.4 (±3.6) years for boys and girls respectively with difference in mean age at marriage of 3.4 which is similar to the national figures for rural areas [17]. Females produced offspring within 2 years of marriage (20.4 ± 3.2 years).

### Migration

Migration data shows that about 2% of the population had migrated to the area (Table 2), mostly as family units, and the majority migrated from adjacent districts, including Dolakha (14.8%), Sindhupalchock (14.5%), Ramechap (12.5%), Kavrepalanchok (6.6%) and Kathmandu (4.9%). On the other hand, out-migration (i.e., predominantly one member per house, who moved for work or study) was 1.36% (Table 2). Among the out-migrant population, about one fifth migrated internationally.

### Mortality

Our survey recorded no deaths among infants or children younger than 5 years of age. One third of all deaths occurred below the average life expectancy (i.e., 64 years) [18]. The crude death rate was 3.9 per 1,000 population per year. Non-communicable diseases (NCDs), including hypertension, diabetes mellitus and cancer, were major causes of death (Tables 3). Based on 2001 census, among total deaths, 2.6%, 1%, 0.6% and 3.6% of deaths were accountable to heart diseases, hypertension, diabetes, and cancer respectively [15].

### Health and health behavior

**Figure 5:**
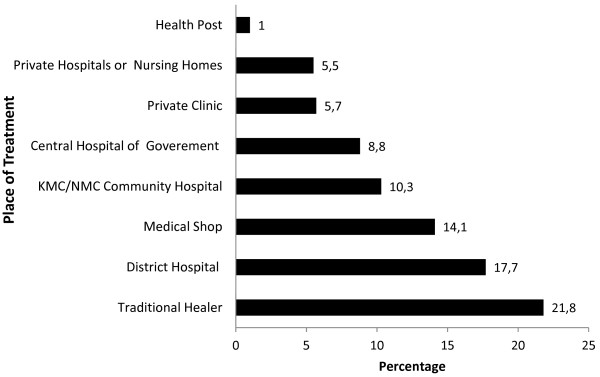
Health service utilization by people during illness, Baseline Survey 2010 (multiple answers).

Nearly 15% of individuals in the study area smoked cigarettes (1 in 5 among the males and 1 in 10 among the females), and about 67% of smokers belonged to an upper-lower class family (data not shown). One in 10 people reported being ill during the four weeks immediately preceding the survey (Table 2). Table 4 shows the top 10 causes of morbidity. The most common cause of illness was respiratory problems, followed by heart disease, hypertension and gastric ailments. Age-adjusted multivariate analysis of the composite prevalence of the main four NCDs (i.e., heart disease, hypertension, cancer and diabetes) shows that NCDs occur more frequently in females, Tibeto-Burman ethnic groups, agricultural workers or laborers, the illiterate and smokers (Table 5) [19]. Nearly 25% of ill individuals visited traditional healers. For modern medicine, they visited the district hospital or bought medicine directly from a medicine shop; they visited local governmental outlets (e.g., health posts and the private hospitals/clinics) less frequently (Figure 5).

**Table 4:** Top ten morbidity reported in JD-HDSS, Bhaktapur, 2010

**Types of morbidity**	**Duwakot**	**Jhaukhel**	**Total**
	** (%)**	** (%)**	** (%)**
	**(N = 608)**	**(N = 909)**	**(N = 1517)**
Respiratory diseases	41.1	42.5	41.9
Fever	34.9	45.2	41.1
Headache, vertigo, and dizziness	18.8	15.4	16.7
Bone and joint pain	8.6	18.3	14.4
Gastrointestinal problems	10.5	15.9	13.9
Heart diseases, including hypertension	13.5	5.6	8.8
Accidents and injuries	4	2.2	2.9
Skin problems	1	4.1	2.9
Diabetes mellitus	5.3	0.9	2.6
Dental problems	1.3	1	1.1

Data is for illnesses within four weeks prior to the survey. Multiple responses were recorded if more than one cause was provided. Respiratory diseases included both chronic obstructive lung diseases and pulmonary infections. Non-communicable diseases such as hypertension and diabetes were self-reported and are prevalent alongside communicable diseases.

**Table 5:** Age-adjusted multivariate analysis for non-communicable diseases

**Variable**	**Number**	**Prevalence**^**#**^	**Adjusted odds ratio**	**95% CI**
**Sex**				
Female	2,702	5.8	1.5	1.1;1.9
Male	2,746	3.9	1	
**Ethnic Group**^**##**^				
Tibeto Burman	2,169	6.6	1.9	1.5;2.4
Indo Aryan	3,152	3.7	1	
**Occupation**				
Agriculture	739	6.1	2.4	1.5;4.0
Labour	229	4.8	2.4	1.2;4.9
Business	368	3.3	1.6	0.8;3.3
Housework	1,559	5.4	2.4	1.5;3.9
Service	1,228	2.1	1	
**Education**				
Illiterate	1,893	7.3	1.67	1.3;2.2
Literate	3,533	3.6	1	
**Smoking**				
Smokers	1,621	34.8	1.09	0.8;1.4
Nonsmokers	3,375	29.8	1	
**Migration**				
Migrated	211	1.9	0.9	0.8;1.0
Native	5,232	5	1	

Results are shown for the population above 30 years of age, (N = 5448)*. Non-communicable diseases included self-reported heart disease, hypertension, cancer and diabetes.

Overall 4.3 3.83 4.86.

^#^Missing cases were excluded from the study.

^##^ Ethnic groups are defined according to Population Monograph of Nepal [19]. Indo Aryan: Brahmins, Chhetris and Thakuri & Tibeto Burman: Magar, Rai, Newar.

### Challenges at JD-HDSS

HDSS provide methods for collecting unique and continuous demographic data that describes an entire population; hence, producing reliable data is both imperative and a major challenge. Our team faced many administrative, political, geographical and social challenges during establishment of the JD-HDSS.

Before performing the baseline survey, our team conducted several rounds of discussion with concerned authorities (i.e., medical schools, governmental bodies, local authorities and political leaders) about the importance of JD-HDSS. One challenge involved justifying the need for the study and then obtaining the commitment of the authorities, which was crucial to the long-term continuation of the project.

Another critical element in establishing a well-defined surveillance site involves hiring appropriate human resources from within the study locality. The norm developed in JD-HDSS involves hiring local data enumerators and supervisors, thus establishing an easy rapport between the study population and the researchers, enhancing convenient data collection and benefiting economic activities. Although the minimum qualification was grade 10 for data enumerators and an Intermediate passed for field supervisors, bachelor or master-level candidates applied for both posts. Thus, high competition and overqualified candidates increased the challenge of selecting suitable manpower. Moreover, political leaders and bureaucrats exerted additional pressure during the recruitment process.

As JD-HDSS is located in mid-hills, participant households were scattered even within wards and they are not easily accessible from the single concrete road of the village. Indeed, access to the entire area is limited by the road facility and the poor availability of public transportation. It requires more than an hour to travel from one location to another. Moreover, Nepal lacks a systematic numbering system for its households, making it very difficult for enumerators and field supervisors to identify the exact location of some houses and, thus, hampering fieldwork.

Household members were not always available during the survey visits, necessitating a second visit. In addition, even when respondents were at home, they were often busy and unable to devote the time required to complete the lengthy questionnaire (9 different parts in 16 pages). Similarly, the questionnaire included the collection of very difficult information regarding migration. Despite these difficulties, our supervisors and enumerators were able to collect information from 96% households.

Data management is a crucial component of reliability and validation. At JD-HDSS, the preparation of data for analysis included several stages. First, completed forms were kept in the JD-HDSS office, where staff members checked that they were filled out completely. Next, the completed forms were sent to the data entry operators. If they found an error, the data entry operators sent the forms back to the field for correction. For example, some forms were completed in mixed language (i.e., both English and Nepali). Frequent cross-checking between computer entries and filled forms ensured accuracy and completeness. This type of scrutiny increased the focus and awareness of the data entry operators.

Similarly, all concerned staff members received guidance on the ethical issues regarding the storage and use of data. All clean data sets were shared among the concerned authorities of JD-HDSS in a format that was user-friendly for scientific publication. Because keeping completed forms and other necessary documents in safe, long-term storage posed another challenge, it was necessary to develop a code of conduct that governed access and sharing of the data.

### Ethical Issues

We encountered many ethical issues during the baseline survey. Although such issues did not reach the sensitivity level of clinical or drug trials, respecting the dignity, feelings, freedom and confidentiality of all respondents was essential. While establishing an HDSS in a selected area, one hurdle involves the fact that many other groups may already have visited the households to collect data for different purposes. Consequently, study participants may become irritated by the attention they receive for monitoring purposes and they may demand immediate direct benefits. Because team members were unable to provide medication to ill participants, such individuals wanted assurance about the long- term benefits of participating in the survey. Team members explained the plan to develop their village as a health model village in Nepal, and households received 25%–50% discounts on hospital services at Kathmandu Medical College and Nepal Medical College.

We also encountered many issues regarding migration. Some participants did not wish to disclose the reasons for their migration (e.g., political pressure, family issues). Similarly, they did not want to share information about monthly income, some diseases (e.g., uterine prolapse), sexually transmitted infections and HIV/AIDS.

Although respondents received no in-hand benefits for participating in the baseline survey, the HDSS team is now considering awarding each household with in-hand benefits (e.g., soap, toothpaste, toothbrushes) during data collection by enumerators and posters, pamphlets and health education when they complete the baseline study. In the long-term, it simplifies the collection of data and also ensures that the project will remain sustainable.

## Discussion

### Relevance of JD-HDSS

This HDSS in progress is the first in Nepal with focus on health issues. A Household Registration System with HDSS methodology had been in the running in the late 1990’s in the Southern Nepalese town of Chitwan in collaboration with Population Studies Centre [1]. However, that project mainly investigated migration issues and followed even those who had out-migrated from the study area after the start of the project.

The intention of this paper is to share the methodology and initial experience of establishment of a HDSS in Nepal and we aim to present longitudinal data after completion of planned subsequent rounds. In terms of the population size, compared to other HDSSs such as Agincourt in South Africa and Matlab in Bangladesh, JD-HDSS is a modest endeavor in terms of the area covered and population size [6,20]. Nonetheless, as put forward by Byass and colleagues, there is no exact formula or statistical ways to determine the optimal sample size of a HDSS [5]. Similarly, the external validity of a study is also an important parameter and in that sense, JD-HDSS is a prototype of the villages near the cities in Nepal which are fast growing into peri-urban or sub-urban stature. However, given the ethnic and geographic diversities of Nepal, it cannot claim to be representative of all the ethnic and geological facets of the country. Nevertheless, from the point of view of study objectives, the selection of HDSS area is arguably well suited because historically establishment of a HDSS has always been tailored to the local needs. The HDSS in Western Kenya, for example, was initiated as a field study site for testing the effectiveness of insecticide-treated nets [10]. In that regard, JD-HDSS is optimal in terms of its current focus on urbanization and NCD risk factors, as the chosen villages are rapidly undergoing socio-developmental transitions. And, as shown by our baseline survey from the HDSS, in its early stage of demographic transition, Nepal faces a double burden of diseases. This is characterized by simultaneous infectious disease and an increasing NCD burden due to urbanization and changing lifestyles [21]. Without preventive measures to combat this trend, Nepal will experience a significantly increased NCD burden. Due to current emphasis on curative care rather than preventive measures, Nepal does not identify the root causes of disease (i.e., hidden social, cultural and demographic aspects). Different cross-sectional studies conducted at various intervals have mentioned these risk factors but failed to establish casual relationships between risk factors and disease [22]. Similarly, Nepal has promulgated laws for registration of vital statistics (i.e., birth, death, marriage, divorce and migration) but has not established the necessary reporting mechanisms yet. Consequently, Nepal needs to establish HDSS to monitor different demographic events: birth; deaths; marriage; migration and health events: types of sickness; hospital visits etc. within a defined geographical community, and also provide a podium for interventional studies regarding subpopulations [8,10].

Nepal has experienced rapid urbanization (i.e., nearly 4.7% per year) [23]. Located near Kathmandu, the capital city, JD-HDSS has already begun the process of shifting from a rural to urban community. Changing lifestyles, from traditional to modern, significantly impact demographic and social structures. Additionally, in- and out-migration impacts JD-HDSS, whose positive net migration rate (0.0085%) indicates population gain. The current baseline survey determined that NCDs are also prevalent alongside communicable diseases, noting that the population, not unexpectedly, has a double burden of disease. Thus, the JD-HDSS study site provides a good platform for studying disease burden from the dual aspects of both health promotion and disease prevention.

### Findings from the JD-HDSS baseline study

The crude birth and death rates of the JD-HDSS are 35% and 53% lower, respectively, compared to national figures (CBR =27.7 per 1,000 population/CDR =9.7 per 1,000 population) estimated in the year 2008 [18]. Total age dependency ratio (younger than 15 years and older than 60 years) was 31 per 100 working-age population (15–60 years), of which 24 per 100 belonged to under 15 years of age. Similarly, the baseline survey observed no mortality in infants, children younger than five years of age, and mothers. According to the Nepal Health Demographic Survey 2011, the national infant and under-five mortality rates are 46 and 54 per 1000 live births, but a wide variation has been noted, for example, according to place of residence (urban and rural rates are 38 and 55, 45 and 64 respectively) [24]. Thus the reason for not finding any child or maternal death could be because of improved living standard in the selected study area, easy access to healthcare services (including access to two community hospitals and a health post), the low illiteracy rate, increased income (only 2.4% lower class), and healthier nutrition. It could as well be because of smaller study population.

The study also determined that more than 90% of children were born in health institutions, more than three times the rate disclosed in the preliminary report of the Nepal Health Demographic Survey 2011, where only 58% of mothers received prenatal care from a doctor or nurse and only 28% of all births occurred in health facilities [25]. Although JD-HDSS has met the Millennium Development Goal (MDG) target (i.e., 60% of all births attended by a skilled provider), Nepal itself is far from attaining that goal [16]. About 33% of deaths occurred in individuals younger than 65 years of age in the study area, about two times less than that reported by the 2001 national census [15]. The census also reported higher life expectancy in Bhaktapur district (71.33 years) compared to the Kathmandu and Lalitpur districts (69.5 years and 67.10 years, respectively) and the entire country (64.1 years) [14].

The net migration rate for JD-HDSS was 0.0085% (in-migration = 2.25; out-migration = 1.36). In-migration increased from 13.4% to 26.8% between 1971 and 2001, respectively. Rural-to-urban migration predominates in Nepal, especially in the large cities of Kathmandu, Lalitpur and Bhaktapur districts. According to national census 2001, those districts received 71.8%, 82.7% and 44.6% in-migrants, respectively [26,27]. The same census showed, the five major reasons for in-migration -business, agriculture, service, education and marriage- mirrored our findings (data not shown) [26,27] . In our study, the majority (91%) of out-migrated people explained that they migrated to increase earning power and get a better education.

The annual health report for fiscal year 2009–2010 listed gastritis, respiratory problems, and injuries among Nepal’s 10 leading morbidities [18]. About 53.22% of the total population visited the outpatient department in Bhaktapur district, of which 57.27% were male. Among them, 9.22% sought outpatient treatment for skin diseases; 6.17% for gastritis; 3.76% for cardiovascular diseases; 3.18% for dental problems, including pain; 3.70% for headache; 2.57% for hypertension and 1.91% for pain [18]. JD-HDSS observed similar morbidity patterns.

In the JD-HDSS study area, NCD prevalence among individuals older than 30 years was 4.32% associated with several factors, including sex, ethnic group, occupation and education. Currently, few community-based studies have investigated NCD risk factors in Nepal. One study, conducted in Eastern Nepal, reported an NCD prevalence rate of 6% in adult males, and a 2008 survey studying NCD reported that more than 80% of respondents exhibit one or more risk factors [28,29]. A recent hospital-based study, conducted in 31 health institutions in Nepal, reported that 36.5% of patients suffer from NCD and share of female was 52.4% of the total patients [30]. Our study showed that smoking carries a 26% risk for developing NCD (unadjusted odds ratio1.26 [95% CI: 0.97–1.63]) compared to 60% reported by a recent hospital-based study in Nepal. Similar to our findings, that study also reported that the adjusted odds ratio for smoking was not statistically significant [30].

Ongoing challenges for NCD control in Nepal include political instability, poor health literacy, greater focus on curative aspects and rapid urbanization [31]. The control approach works well if lifestyle factors are modified through effective health education intervention and tobacco control policies. To forecast cumulative risk, future studies should investigate risk factor clusters (i.e., a combination of two or more risk factors) for NCD development rather than focus on single-factor risks [32].

### Limitations

Although our success in establishing Nepal’s first HDSS provides a reliable sampling framework for subordinate studies and builds mutual trust between village residents and researchers, the baseline survey includes several limitations that require further attention. Because we obtained our baseline data from any family member who was older than 18 years of age, our data may be both over- and under-reported. There is also a possibility of bias regarding both recall and selection. Selection bias may arise due to the selection of convenient respondents from whom the enumerators can obtain information easily, whereas supervision bias may result from time constraints regarding form checking. Because the JD-HDSS geographic area was selected purposively, our survey covered only two village developmental committees; hence, the findings should be generalized cautiously to populations beyond the surveyed population [33].

To elicit information on death and its cause, the survey interviewer asked the question “Has any member of your family died within the year?” If the answer was yes, respondents were asked about age, sex and the cause of death. So there is possibility of measurement bias, e.g., data enumerators might fail to record all deaths that lead to under reporting of deaths and hence the possibility why the crude death rate in the JD-HDSS is below the national figure (8.3 per 1,000 population). Also, the emotional attachment with the death of a family member may make the respondents not wanting to talk about it. Furthermore, the enumerators do not ask for any details regarding deaths. In addition, there is the possibility of recall bias, i.e., the respondents cannot remember exact date of deaths.

We also found that only 7 of 10 deaths were reported to the village office. It indicates that the vital registration system does not cover all deaths and fails to provide accurate data. This underlines the importance and need of an HDSS in Nepal, which enables recording of deaths on a regular basis. Admittedly, the verbal autopsy method would have been a better tool to elicit the causes of deaths [34]. We were unable to use verbal autopsy for the baseline study because of lack of trained manpower but do intend to use them in future surveys.

Additional interviewer bias can also come from the enumerators as many of them were overqualified for enumeration work and they can add their own thoughts in to a response while recording it. Similarly, the response to the enumerators belonging to a different political ideology can hamper the respondents’ answer during the interview. Additionally, the length of the questionnaire may have contributed hurried and often untrue or incomplete responses. Similarly, lack of ‘on-the-spot’ benefit could have biased the respondent’s reply.

Finally, provision of ancillary care is an ethical dilemma for which there are no clear-cut guidelines or solutions [35]. Over-inflation of the HDSS budget is an important possibility to consider while providing such care [36].

### Strengthening of the JD-HDSS

We have used basic technology for village mapping and data collection in the baseline census. Mapping can be done more scientifically in the future with the use of GPS technology like in FilaBavi and many African HDSSs [9,10]. This could also have paved way for mapping of the environment coverage of the HDSS [37]. Cardiff Teleforms, used for example in Western Kenya, could also be helpful if there is possibility of technical support [10]. Even data entry and analysis software can be upgraded to maintain and produce quality data [6]. Effort should also be made towards improving the internal validity of our data collection technique by using more widely accepted methods such as Verbal Autopsy for information on cause of mortality [6,38]. Expansion of the study area itself to include a more urban area can yield interesting comparisons in demographic and health parameters [39]. Finally, effort should be made to eventually make this HDSS an IN-DEPTH member.

### Future studies

A new round of census survey will begin in September 2012, followed by another survey every six months. JD-HDSS is also currently conducting two longitudinal subordinate studies, one related to tobacco smoking behavior among 500 adolescents and the other related to knowledge, attitudes and practice of cardiovascular disease in people aged 25–59 years. Additional subordinate studies on maternal health and nutritional status are in the pipeline.

In agreement with the thoughts put forward by Baiden, the JD-HDSS can be a good field site for other scholarly interests as the project is primarily of academic nature [40]. Presence of gaps in socio-economic parameters and diversity in health-care utilization, studies on issues such as socio-economic determinants of health, health equity and health system reform are interesting tentative projects in the JD-HDSS [41-43]. As done in the Agincourt HDSS, triangulation of our data with the relevant national data could also be a potentially useful exercise particularly in the context of urbanization and its complexities [44].

## Conclusions

In tandem with the current baseline survey, the study reported here demonstrates that it is possible to establish an HDSS in a village area of Nepal to collect accurate and reliable data. Such prospective data on health and demography also provides a platform for public health research and the evaluation of community-based health policies and practices. Results from the baseline survey show that both maternal and child health in the surveillance site exceed that of the entire country. The baseline survey also reported on risk factors associated with NCDs. Thus, studying NCD burden in the HDSS can yield reliable epidemiological data for prevention and control through longitudinal studies.

## Competing interests

The author(s) declare that they have no competing interests.

## Authors’ contributions

URA, AV, and SSV carried out the research. URA and AV equally contributed to the study and data analyses and wrote the majority of the paper. SSV contributed to the manuscript draft. MP and AK participated in the design of the study, data analyses, and supervision and helped draft the manuscript. All authors have read and approved the final manuscript.
